# Cellular Prion Protein Role in Cancer Biology: Is It A Potential Therapeutic Target?

**DOI:** 10.3390/biomedicines10112833

**Published:** 2022-11-07

**Authors:** Saba Yousaf, Muhammad Ahmad, Siwen Wu, Muhammad Anjum Zia, Ishtiaq Ahmed, Hafiz M. N. Iqbal, Qingyou Liu, Saif ur Rehman

**Affiliations:** 1 State Key Laboratory for Conservation and Utilization of Subtropical Agro-Bioresources of Guangxi University, Nanning 530005, China; 2 Enzyme Biotechnology Laboratory, Department of Biochemistry, University of Agriculture Faisalabad, Faisalabad 38040, Pakistan; 3 Faculty of Veterinary Sciences, Shaheed Benazir Bhutto University of Veterinary and Animal Sciences (SBBUVAS), Sakrand 67210, Pakistan; 4 Department of Regional Science Operations, La Trobe Rural Health School, Albury-Wodonga, VIC 3689, Australia; 5 Tecnologico de Monterrey, School of Engineering and Sciences, Monterrey 64849, Mexico

**Keywords:** prion, prion protein, cancer biology, PRNP, ERK2 (MAPK1) pathway, EP/MAPK pathway, cancer cell drug resistance, therapeutic target

## Abstract

Cancers are worldwide health concerns, whether they are sporadic or hereditary. The fundamental mechanism that causes somatic or oncogenic mutations and ultimately aids cancer development is still unknown. However, mammalian cells with protein-only somatic inheritance may also contribute to cancerous malignancies. Emerging data from a recent study show that prion-like proteins and prions (PrP^C^) are crucial entities that have a functional role in developing neurological disorders and cancer. Furthermore, excessive PrP^C^ expression profiling has also been detected in non-neuronal tissues, such as the lymphoid cells, kidney, GIT, lung, muscle, and mammary glands. PrP^C^ expression is strongly linked with the proliferation and metastasis of pancreatic, prostate, colorectal, and breast malignancies. Similarly, experimental investigation presented that the PrP^C^ expression, including the prion protein-coding gene (PRNP) and p53 ag are directly associated with tumorigenicity and metastasis (tumor suppressor gene). The ERK2 (extracellular signal-regulated kinase) pathway also confers a robust metastatic capability for PrP^C^-induced epithelial to mesenchymal transition. Additionally, prions could alter the epigenetic regulation of genes and overactive the mitogen-activated protein kinase (MAPK) signaling pathway, which promotes the development of cancer in humans. Protein overexpression or suppression caused by a prion and prion-like proteins has also been linked to oncogenesis and metastasis. Meanwhile, additional studies have discovered resistance to therapeutic targets, highlighting the significance of protein expression levels as potential diagnostic indicators and therapeutic targets.

## 1. Introduction

**Figure 1:**
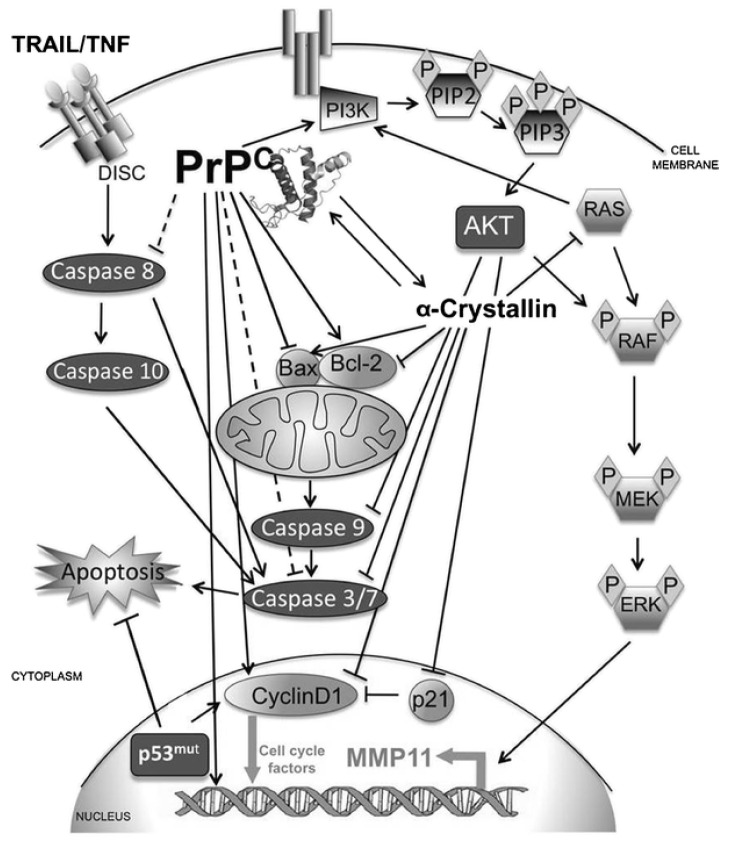
Schematic diagram showing the molecular interactions of the cellular prion protein (PrP^C^). Reprinted from Ref. [7] with permission from Springer Nature. License Number: 5383991150266.

PrP^C^, the prion protein cellular form, shares 90% of its amino acid sequence with other mammalian proteins. PrP^C^s are expressed almost in all tissues of an organism, but a higher amount of PrP^C^s has been found in the central nervous system (CNS), particularly the synaptic membranes and PrP^C^ are linked. Scrapie PrP (PrPSc), a mutant cellular prion protein with an altered structure, is assumed to be the key etiological cause of prion diseases. The PrP^C^ and PrPSc could interact with each other using α-helices present in PrP^C^ and β-sheets of the PrPSc while the precise method of this transformation is still not fully known. Prion is a protein constituting infectious particles that leads to a variety of neurodegenerative diseases as well as cancer. There are two isoforms of the prion protein: the cellular prion protein (PrP^C^) and the scrapie prion protein (PrPSc) [1]. However, the precise role and mechanism of PrPSc are unknown. This study intends to shed light on PrPSc, the underlying cause of deadly illnesses, such as cancer and neurological disorders. In this reverence, there is a dire need to improve the survival rates, and considerable attention is required. PrP^C^ can be exploited as a therapeutic target in cancer therapy. The glycosylphosphatidylinositol (GPI) is an anchored protein attached to the membrane of prion protein (PrP^C^) and the expression of PrP^C^ is initiated during embryogenesis and maximum, during puberty. Cellular prion protein is present in peripheral organs, neurons and the nervous system [2]. PrPSc can cause disease (neurodegenerative disorders) in a certain way that normal prion protein found on cell surfaces behave abnormally (i.e., misfolding) and accumulate in the brain, which subsequently results in brain damage and neurodegenerative disorders like Creutzfeldt Jakob disease [3]. PrP^C^ altered expression is involved in cancer progression, and PrPSc (i.e., altered variant) misfolded form is responsible for causing neurodegenerative diseases. More extensive work is required to rule out the exact mechanism of disease progression. Prions are normally distributed in the whole body of an organism, but disease-causing prions are distinguished in their structure and composition. PrP^C^ has a lipid chain of GPI attached to its structure and is present in ample amounts in CNS [4]. In humans, prion protein (PrP) is encoded by the PRNP gene that is present on the 20th chromosome. Previously, it was assumed that PrP^C^ primarily affects the CNS only. However, it is now found in other non-neuronal tissues like lungs, heart, kidneys, GIT, muscles, lymphoid cells, mammary glands, etc. [5]. It was reported that PrP^C^ also participates in causing incurable diseases like breast carcinoma, gastric cancer, prostate cancer, and glioblastoma [6]. PrP^C^ also boosts cancer cell proliferation, tumorigenesis, and G1/S transitions in cancer. In addition, PrP^C^ introduces helpers to complete its function in the signaling pathways [7]. The molecular interactions of the cellular prion protein (PrP) is shown in Figure 1 [7]. Thus, PrP^C^ is a pathologically aggregated prion protein involved in neurodegenerative disorders [8].

PrP plays a significant role in the progression of various neurodegenerative disorders and cancer, but the precise pattern of their action is still unknown. The fluctuations in PrP^C^ expression pattern (either increased or decreased) might cause the development of various malignancies such as the PrPSc misfolding could result in severe catastrophic neurological diseases. Previously it was thought that PrP^C^ is primarily involved in neurological illnesses only, but recent research has revealed that it is also a hidden cause of cancer growth. The protein-only model of prion propagation was first developed using a yeast prion. Yeast is at the forefront of study in understanding cellular regulation of prion propagation, prion structure, de novo prion synthesis methods, the sensitivity of prion transfer, and biological activities.

## 2. Fungal Prions

In lower eukaryotes, prions and protein-only inheritance have received much attention (e.g., yeast). Some cellular proteins undergo structural metamorphosis into amyloid prions, causing yeast phenotypes transformations [9]. The production of amyloid prion ensures that any further copies of the polypeptide are changed into amyloid prion and passed on to daughter cells. This gives rise to the term “yeast prions” for the amyloid forms among these yeast proteins [10]. The adaptive role that yeasts prions perform in phenotypic inheritance is unrelated to diseases [11]. There are at least 25 prion-forming domains (PrDs) among 6000 proteins that the yeast genome has been revealed to encode, and only 9 of these proteins have been demonstrated to be capable of maintaining the prion structure in their native form [12]. Despite the discrepancies in opinion among different research groups regarding the amino acid composition of known yeast PrDs, the true amount of prion-forming protein in the yeast proteome may be much larger [13]. PrDs, structurally related domains of amyloid prions that allow for structural duality, can be used to make new prions by incorporating them as modules into other proteins. The majority of yeast prions are self-replicating [6].

The most studied yeast prions are [*PSI*^+^] prion yeast of *Saccharomyces cerevisiae* and [*PS1*^+^] is the prion state of the translation termination factor eRF3 (Eukaryotic Polypeptide Chain Release Factor 3), also known as Sup35p (eukaryotic translation release factor of *S. cerevisiae*) in yeast. The function of Sup35p is reduced when it is converted to the [*PSI*^+^] prion, resulting in a faulty process of termination of translation. Thus, proofreading of mRNA termination codons occurs as a result of the prion form. It was thought that [*PSI*^+^] generated polypeptides with altered functionalities and longer C-termini, which increased yeast phenotypic plasticity and its ability to withstand challenging environments [11]. The functional conformational alterations in polypeptides and the prolonged C-terminal modifications are similar to “cancer-like” changes. To determine whether a strain is [*PSI*^+^], stop codons caused by nonsense mutations that occur in [*PSI*^+^] strains are read through. This serves as an example of nonsense mutation suppression [14]. The Sup35p’s ability to manufacture prion has survived years of evolution. However, [*PSI*^+^] prion is not found in yeast isolates, and some [PSI^+^] isoforms are highly cytotoxic [9]. The Sup35p possesses a PrD at the N-terminus, like other yeast prion proteins. The PrDs of Sup35p as well as other yeast prions have an unusual amino acid profile with higher polar repeats and low in charged residues, resulting in a molten globule-like protein form that favors the formation of amyloid-nucleating junctions. Prionization and determining the pathogenic characteristics of prion proteins depend on PrDs [15].

**Figure 2:**
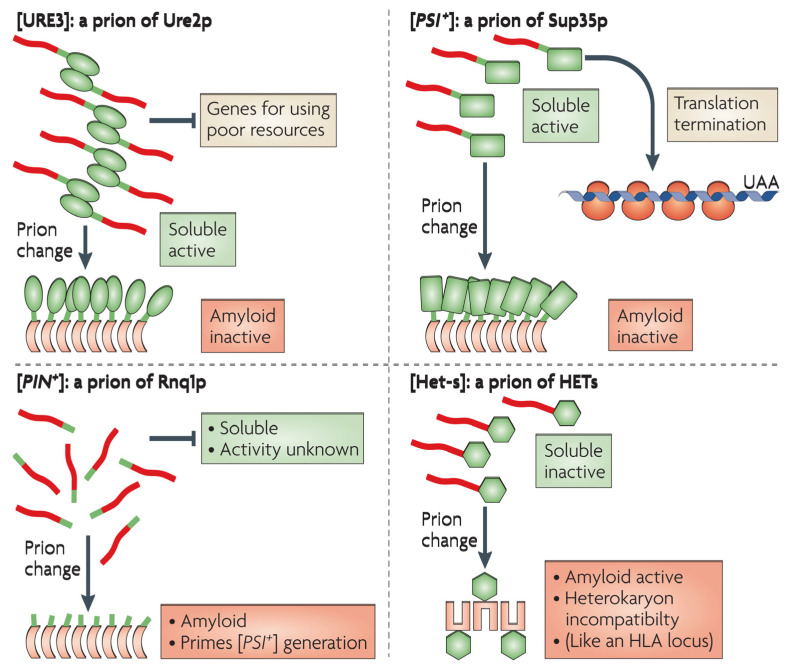
Illustrations of yeast and fungal based amyloid prions. The natively structured domains are shown as green shapes. Whereas the unstructured domains in native form and later become amyloid in prion form are shown as red shapes. For a detailed description, see Ref. [16]. Reprinted from Ref. [16] with permission from Springer Nature. License Number: 5384000379309.

The yeast genome can encode several prion-forming domains, which led the prion proteins to exist in multiple variants or phenotypes. The majority of yeast prions are self-replicating as shown in Figure 2 [16,17]. The non-chromosomal genetic component of [URE3] is an infectious form (prion) of the URE2 protein and appears to be self-replicating amyloidosis. Protein chaperones facilitate the propagation of [URE3], demonstrating that protein structure influences heredity.

## 3. Boosts Proliferation of Cancer Cells

PrP^C^ has the ability to stimulate cancer cell growth and in gastric cancer cells, the overexpression of PrP^C^ was found to be associated with cell proliferation by activating the phosphatidylinositide 3-kinase (PI3K) pathway and promoting the G1/S phase transition by upregulating cyclin D1 [18]. The transition from the G1 to S phase is likewise related to PrP^C^. In HT29 (cell line for human colorectal adenocarcinoma) colon cancer cells, knocking down PrP^C^ decreased the cell growth and increased the inhibitory effect of fucoidan by reducing the production of cyclins and cyclin-dependent kinase (CDK) [19]. In glioblastoma (GBM) cells, the PrP^C^ interaction with the co-chaperone Hsp70/90 organizing protein (HOP) could boost the proliferation via activating the PI3K and extracellular signal-regulated kinase (ERK1/2) pathways [20]. Additionally, the HOP and PrP^C^ interaction might increase the propagation of glioma stem-like cells, while the decreased expressions of PrP^C^ suggested that HOP could be used as a GBM therapy [21]. Cancer cells prefer to produce their energy by aerobic glycolysis; therefore, this leads to a reduction in the available energy for synthesis, cell survival, and proliferation [22]. The overexpression of PrP^C^ could also induce the Warburg effect in colorectal cancer cells by up-regulating glucose transporter 1 (Glut1) expression, which in turn enhances the absorption of glucose via epigenetic regulation of the Fyn-HIF-2a-Glut1 pathway [23]. Moreover, it also interacted with Notch1 to promote proliferation in pancreatic ductal adenocarcinoma (PDAC) [24]. PrP^C^ stimulates the ERK1/2 and PI3K/protein kinase B (AKT) signaling pathways that promote cell proliferation in schwannoma cells when it binds with the 37/67 kDa non-integrin laminin receptor. In this way, prion protein interrupts different signaling pathways and metabolic cycles, altering normal biosynthetic processes thereby promoting cancer cell invasion and growth.

## 4. PrP^C^ Encourages Cancer Cells to Invade and Spread

**Figure 3:**
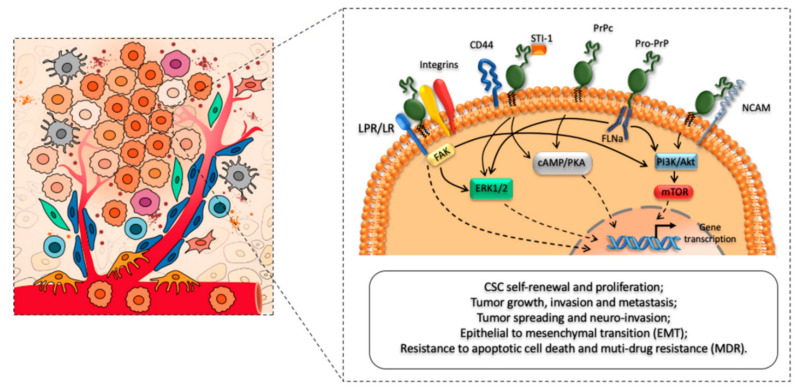
Schematic illustration of the regulatory role of PrP^C^ in promoting cancer stem cells (CSCs) self-renewal, proliferation, and migration patterns. For a detailed description of CSCs phenotype, biology, functioning, and cross-talk between CSCs and major cellular components of the tumor micro-environment (TME), see Ref. [16]. Reprinted from Ref. [28] with permission under the terms and conditions of the Creative Commons Attribution (CC BY) license.

Over 90 percent of the total cancer-related deaths are caused by metastasis, although the mechanisms which aid the phenomena require further exploration. The metastatic process is generally categorized into two stages. In the initial phase, the cancerous cells physically move from one location to another [25], whereas the second stage involves the initial tumor spreading to additional distant tissues. Cancer cells that have spread colonize their new surroundings. Epithelial-to-mesenchymal transition-in-between is referred to as EMT [26] and demonstrated to be significantly involved in several in vitro models. The PrP gene expression dramatically increased during EMT and the upregulated PrP^C^ let the EMT-like cells to be undifferentiated. This characteristic has also been identified in invasive colorectal cancer cells (CRC) [27]. Most details about the EMT amplification in PrP^C^ mechanics are unclear [28]. Moreover, the SATB1 (special AT-rich sequence-binding protein 1) is a nuclear matrix-associated protein. Tumor metastasis can be characterized through chromatin structure modifications, up-regulating the metastasis-associated genes, and down-regulation of the tumor-suppressor genes [29]. Fyn and specificity protein 1 (SP1) played a significant role in the reduced expression of SATB1 and tumor progression in CRC following PRNP knockdown. The PrP^C^-Fyn-SP1-SATB1 axis may be up-regulated by PrP^C^, which would support tumor metastasis [30]. Moreover, overexpression of g-Syn and PrP^C^ is also found in CRC. They might contribute to the spread of colorectal cancer cells by inducing an inflammatory response [31]. While the increased expression of PrP^C^ in metastatic gastric cancer cells can endorse the invasion and metastasis by activating the mitogen-activated protein kinases (MEK)/ERK pathway, which leads to matrix metalloproteinase-11 transactivation (MMP11) [32]. The MMP11 can aid in tissue remodeling, inflammation, and matrix breakdown. The signal that PrP^C^ uses to encourage invasion must be sent by its N-terminal section [33]. A type 1 endogenous matrix metalloproteinase inhibitor (MT1-MMP) is a tissue inhibitor of metalloprotease (TIMP) [34] and TIMP adhesion to the prion protein GPI anchor resulted in a membrane-bound, high-affinity designer TIMP that is exhibited on the cell surface and co-localized with cellular MTI-MMP. As depicted in Figure 3, PrP^C^ as a main regulator of CSCs phenotype, biology, and functioning [28].

PrP^C^ has been shown to boost EMT in colorectal cancer stem cells by activating the ERK2/(MAPK1) pathway. Thus, providing affirmed bases that the morphology of CSCs (cancer stem cells) and EMT are directly interlinked. Notch1 could influence the CSCs [18]. Both the CSC and EMT are co-localized on the cellular membrane and act as the downstream effectors of the PrP^C^ that promote the metastasis of pancreatic cancer cells [35]. Through the down-regulation of PrP^C^-Oct4 pathways, 5-fluorouracil (5-FU) and melatonin can lower the octamer-binding transcription factor 4 (Oct4) markers for colon CSCs [36]. This method will probably prevent cancer from spreading by decreasing tumor-mediated angiogenesis [37]. PrP^C^-containing exosomes released by CRC may promote tumor growth even further by alleviating the body’s amount of cellular prion protein [38]. The anti-PrP^C^ and 5-FU combination also slow down the growth of tumors. The immune system is one of the most important systems for halting the emergence and development of cancer [39]. One of the key targets of cancer immunotherapy is the regulatory T cells (Tregs), which suppress the immune response. Increased PrP^C^ expression stimulates the proliferation of regulatory T cells by up-regulating the altered growth factor-beta (TGF-b) and programmed death ligand-1 (PD-L1), which speeds up the progression of the tumor, according to a lung metastatic melanoma model in Prnp0/0 and Tga20 animals. Moreover, it was also reported that PrP^C^ expression encourages cancer cell spread [6]. However, one study reported that knocking down PRNP in mesenchymal embryonic mice cells with Ras/Myc transformation increased the likelihood of lung metastasis. Meanwhile, to fully understand the role of PrP^C^ in cancer spread, further molecular mechanism-based modeled research is required.

## 5. Cellular Pathway

**Figure 4:**
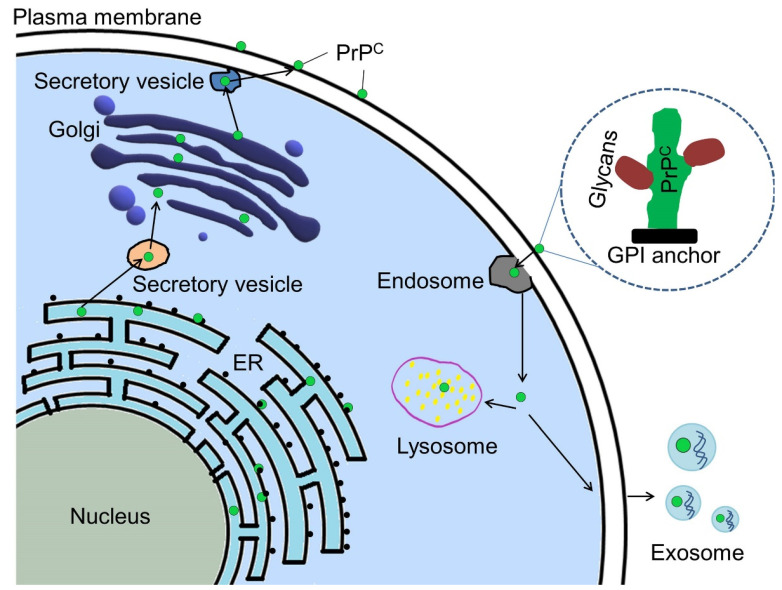
Mechanistic insight and trafficking routes of PrP^C^ within a cellular environment. Green dots are representative form of PrP^C^. See Ref. [43] for a detailed description. Reprinted from Ref. [43] with permission under the terms of the Creative Commons Attribution License (CC BY).

PrP^C^ acts as a scaffold on the cell surface and attracts many other cellular entities, such as proteins which help to carry out its functional roles in signaling pathways [40]. PrP^C^ shares a common biosynthesis pathway with other proteins secreted and bound to membranes. It is synthesized in ribosomes associated with the endoplasmic reticulum (ER), then imported to the ER, where it is glycosylated and modified by the GPI anchor and then delivered to the Golgi bodies for additional modification [41]. The PrP^C^ is then transported [42] to the surface of the cell and absorbed through the endocytic pathway. Incorporated PrP^C^ can either be carried to the lysosome for breakdown or encapsulated in exosomes and excreted outside the cell. The GPI anchor is located at the C-terminus of PrP^C^ and mostly can bind to the lipids of the cell membrane surface. It is also distributed in the cytoplasm and nucleus. PrP^C^ was unexpectedly identified in cancer cell-produced exosomes as shown in Figure 4 [43].

The PrP^C^ synthesized by the RER and the GPI anchor modifications along with glycosylation was done to PrP^C^ in the ER before it is transported to the Golgi body for additional modifications [2]. The GPI (glycosylphosphatidyl-inositol) anchored on the PrP^C^ allow it to be detected at the plasma membrane when it has fully matured [44]. Some mature PrP^C^ may be exocytosed and ejected from the cell [45] or endocytosed for lysosome degradation.

Cancer is a phenomenon of worldwide concern and is categorized as the second largest cause of human death around the globe. Recently, researchers have suggested that PrP^C^ is involved in several biological processes related to cancer, including cancer stem cells, metastasis, cell death, and cell proliferation [46,47].

## 6. PrP^C^ Fosters Drug Resistance in Cancer Cells

**Figure 5:**
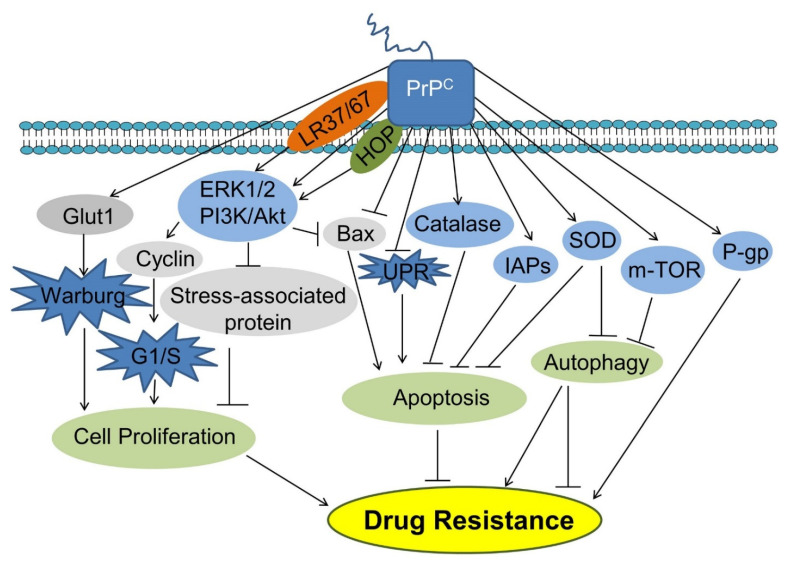
PrP^C^ pathways to cancer cell drug resistance mechanism by stimulating cell proliferation and inhibiting apoptosis. Abbreviations: HOP, Hsp70/90 organizing protein; IAPs, Inhibitors of apoptosis proteins; Glut1, Glucose transporter 1; PI3K, Phosphatidylinositide 3-kinase; AKT, Protein kinase B; Bax, Bcl-2-associated X protein; UPR, Unfolded protein response; SOD, Superoxide dismutase; P-gp, P-glycoprotein. Reprinted from Ref. [43] with permission under the terms of the Creative Commons Attribution License (CC BY).

Drug resistance predominantly antibiotics resistance is a crucial aspect in cancer treatment and can be caused by a variety of reasons [48]. Multi-drug resistance (MDR) and cell death inhibition are two important concerned mechanisms explaining the PrP^C^ participation in developing drug resistance while treating cancer. The ability of a cancer cell to resist a variety of anti-cancer medications is referred to as multi-drug resistance (MDR) [49]. Necrosis, autophagic cell death, and apoptosis are the three main kinds of cell death (Type I programmed cell death). Apoptosis is characterized by DNA cleavage, chromatin condensation, membrane protrusions, cell shrinkage, and caspase activation [50]. A lysosomal intracellular destruction pathway called excessive autophagy, which is a kind of autophagy, results in autophagic cell death as shown in Figure 5 [43].

Necrosis (non-programmed cell death), which is brought on by unforeseen impacts on the cells, is characterized by plasma membrane rupture accompanied by cytoplasmic leakage [32]. In a variety of cancer cell types, PrP^C^ up-regulation can lead to treatment resistance. PrP^C^ increases the production of cell cycle-associated proteins, which in turn encourages growth and survival in colorectal cancer cells [51]. PrP^C^ also stimulates the PI3K-Akt signaling pathway. Similarly, by up-regulating the inhibitors of apoptosis proteins, PrP^C^ overexpression enabled colorectal cancer LS174T cells more resilient to doxorubicin-induced apoptosis (IAPs) [52]. When PrP^C^ is up-regulated, oxaliplatin resistance is brought on by an increase in superoxide dismutase (SOD) and catalase activity, and also a decrease in endoplasmic reticulum stress and apoptosis [53]. PrP^C^ interferes with the signaling cascade and apoptotic inhibitors, preventing cancer cell death by particular medicines. The phenomenon of drug resistance in several types of cancer is a crucial challenge.

## 7. Pattern of Drug Resistance

PrP^C^ causes treatment resistance in gastric cancer cells through employing various types of mechanisms. PrP^C^ collaborates with MGr1-Ag/37LRP to induce MDR in gastric cancer cells by inhibiting apoptosis via the PI3K/AKT signaling pathway [54]. PrP^C^ peptides may be involved in MDR in gastric cancer via increasing anti-oxidant enzyme activity. PrP^C^ promotes MDR by raising the level of multi-drug resistant protein P-gp (P-glycoprotein) and decreasing apoptosis in breast and gastric cancer cells [55]. PrP^C^ overexpression decreases the Bcl-2-associated X protein (Bax) expression in renal adenocarcinoma ACHN cells while increase the resistance to TNF-induced apoptosis. Exosomes, which are synthesized by the cells and carry PrP^C^ into the surface of the cell by sticking to a cell membrane, are formed by the cells [7]. Doxorubicin binds with PrP^C^ released by the tumor microenvironment, preventing it from entering the nucleus and integrating itself into DNA, ultimately leading to cell death. Patients with high serum PrP^C^ levels who receive doxorubicin treatment are therefore more likely to experience a relapse [14]. PrP, a synthetic peptide derived from human prion protein, which protects schwannoma cells from Hydrogen peroxide-induced cell death, contains amino acids 105 to 120. The anti-apoptotic and anti-autophagic effects of PrPC have been demonstrated in cancer cells. PrP^C^ prevents cancer cells and neurons from undergoing apoptosis [56]. PrP^C^ overexpression prevents the apoptosis that is induced by the expression of Bax, serum deprivation, and anticancer drug treatments. Apoptosis is also inhibited by PrP^C^ when it forms a dimer at its C-terminus with the anti-apoptotic protein Bcl-2 [57]. The Bcl-2/Bax ratio rises with the increase of PrP^C^ levels limiting the MCF-7 breast cancer cells from going through apoptosis. Tumor necrosis factor-related apoptosis-inducing ligand, or TRAIL, is a death receptor ligand that induces apoptosis in cancer cells [58]. When the Bax/Bcl-2 ratio is elevated and PrP^C^ is down-regulated, apoptosis mediated by TRAIL is more likely to occur in adriamycin resilient human breast cancer cells. In hypoxic human colon cancer cells, PrP^C^ prevented TRAIL-induced apoptosis [59]. Furthermore, PrP^C^ could also reduce TRAIL-induced apoptosis by activating Akt. Additionally, PrP^C^ stimulated the PI3K/Akt signaling pathway, which supports PrP^C^’s anti-Bax activity and prevents the early activation-stage Bax pro-apoptotic conformational alterations [43]. Additionally, PrP^C^ decreased the unfolded protein response, which averted lung and pancreatic cancer cells from dying (UPR) [60]. The catabolic process known as autophagy, which occurs in eukaryotic cells, is evolutionarily conserved and involves the digestion and recycling of undesired or dysfunctional cytosolic components through lysosomes [61]. During autophagy, a phagophore surrounds cytosolic elements (cargos), expanding and encapsulating to form the recognizable double-membraned autophagosome. In conditions like famine, the autolysosome that develops when the autophagosome and the lysosome join will break down cargo to produce small molecules which can be used for biosynthesis or energy production for cell viability [62]. Cells may perish when autophagy is overdone (also known as autophagy-induced or autophagic cell death). PrP^C^ can control autophagic cell death in glial tumor cells [63]. It was found that PrP^C^ silencing could inhibit the activity of the mTOR kinase, boosting autophagy and autophagic cell death in T98G glioma cells [64]. Additionally, SOD (Superoxide dismutase), an antioxidant enzyme, was stimulated by PrP^C^, preventing autophagy. PrP^C^ may be able to combat resistance to antibiotics in cancer cells by preventing autophagy because it is largely a pro-cell survival process. Thus, researchers could figure out a strategy to circumvent the obstacle by targeting the pathways influenced by prion protein interaction, which leads to drug therapy resistance and also allow for the development of more efficient and effective site-directed cancer treatment.

## 8. PrP^C^ as a Potential Cancer Biomarker

In a high-resolution cell membrane proteome analysis, PrP^C^ was identified as a potential biomarker for the progression of colorectal adenoma to carcinoma. PrP^C^ was able to differentiate between patients with early-stage colorectal cancer and normal colon tissue as well as high-risk and low-risk adenomas [59]. Several lines of evidence have suggested the possible functioning of PrP^C^s in modulating cancer susceptibility to chemotherapy and, as a result, monitoring the therapeutic efficacy and patient prognosis [65]. Individuals with higher PrP^C^ expression exhibited better therapeutic tolerance, worsening 2-year survival, and a high fatality rate compared to those with lower PrP^C^ expression [66]. As a result, PrP^C^ overexpression in estrogen receptor (ER)-negative breast cancer patients is linked to decreased chemotherapy sensitivity, suggesting that PrP^C^ could be a predictor of adjuvant chemotherapy benefit in ER-negative patients [36]. PrP^C^ expression in human PDAC biopsies is associated with a worse prognosis than in PrP-negative cases, demonstrating that PrP^C^ plays a vital tumor-promoting role in Pancreatic ductal adenocarcinoma (PDAC) [67]. Surprisingly, western blot and immunohistochemical examinations of surgically excised PDAC specimens reveal a significant difference between PDAC and control tissues [9]. PrP^C^ is overexpressed very selectively within the ductal compartment in PDAC specimens, whereas normal control tissues only have a few ductal epithelial cells with moderate PrP staining [68]. While, the PrP^C^ expression was not affected by the presence of dysplastic areas around the “healthy” pancreas, implying that PrP^C^ levels are linked to tumor invasiveness and aggressiveness rather than preneoplastic lesions. However, further trials are needed to fully establish the predictive usefulness of PrP^C^ discovery [69]. Prion protein even resists cancer cells’ response to pharmaceutical treatment. PrP^C^ may alter the susceptibility of cancer to chemotherapy, which would allow it to be used to track the treatment’s effectiveness and patient prognosis.

## 9. Targeting PrP^C^ as a Cancer Treatment

Numerous circumstantial evidence pointed the possibility that prion proteins could result in protein-only inheritance in the setting of the onset and development of cancer. It is hypothesized that the transformation of a regular cellular protein into a prion and the accompanying metabolic disturbance may increase the characteristics of cancer due to the overexpression of prion proteins and their effects on apoptosis, kinase signaling, and chaperone sequestration [4,43]. Infecting prions, like PrPSc, can spread to nearby cells via exosomes implying that prion introduction may aid in developing the cancer phenotype. Prior to the development of genetic mutations, this can encourage the rapid emergence of phenotypic heterogeneity, including chemotherapeutic tolerance, cancer spread, and metastasis. Therefore, it is imperative to ascertain the function of PrP^C^ itself in cancer as well as prions that target PrP^C^ (like PrPSc) and perhaps additional prions that have not yet been identified but may have an impact on other cellular signaling proteins that ultimately affect cancer [31,68,69]. Thus, available genetics or theoretical information could be used to develop possible plans for better prognostication, early detection, therapeutic intervention, and prevention of prion-dependent diseases, including various cancer types [4,36,43,48,60]. The data acquired could be used to develop prospective strategies to improve prognosis, early diagnosis, therapeutic intervention, and prevention of prion-dependent disorders, such as different cancer varieties. Prion protein has also been involved in the proliferation of carcinoma cells, which can be fatal.

## 10. Conclusions

In an organism, there are two distinct forms of PrP: PrP^C^ and PrPSc. Neurodegenerative disorders can be severely debilitating when PrP^C^ is misfolded or aggregates. Recent research has indicated that it may also contribute to cancer. Increased or decreased PrPc expressions also became a secret cause of several malignancies. PrP^C^ encourages the growth of cancer stem cells, drug resistance, invasion and metastasis of cancer cells, and other processes that might speed up the evolution of cancer. As a result, targeting PrP^C^ as a cancer treatment is a novel approach. It is critical to understand how PrP^C^ regularly works to treat prion disorders. The majority of current therapeutic approaches focus on preventing the production of PrPSc. Targeting the cellular pathways that PrP^C^ uses to regulate biological activity might be an additional strategy if changes in PrP^C^ function significantly contribute to prion-induced disease. It is now possible to create in vitro assays that search for medicines with therapeutic promise using the physiological activity of PrP^C^. The barriers in this study are to elucidate all the isoforms of prion proteins and their way of emergence and role in several diseases. Then we can treat diseases like cancer by targeting prions as therapeutic targets.

## Data Availability

Not applicable.
